# Three‐dimensional assessment of impingement risk in geometrically parameterised hips compared with clinical measures

**DOI:** 10.1002/cnm.2867

**Published:** 2017-04-05

**Authors:** Robert J. Cooper, Marlène Mengoni, Dawn Groves, Sophie Williams, Marcus J.K. Bankes, Philip Robinson, Alison C. Jones

**Affiliations:** ^1^ Institute of Medical and Biological Engineering, School of Mechanical Engineering University of Leeds Leeds LS2 9JT UK; ^2^ Guy's Hospital Great Maze Pond London SE1 9RT UK; ^3^ Leeds Musculoskeletal Biomedical Research Unit Chapel Allerton Hospital Leeds LS7 4SA UK

**Keywords:** alpha angle, femoroacetabular impingement, geometric parameterisation, hip joint, parametric model, radiographic measures

## Abstract

Abnormal bony morphology is a factor implicated in hip joint soft tissue damage and an increased lifetime risk of osteoarthritis. Standard 2‐dimensional radiographic measurements for diagnosis of hip deformities, such as cam deformities on the femoral neck, do not capture the full joint geometry and are not indicative of symptomatic damage.

In this study, a 3‐dimensional geometric parameterisation system was developed to capture key variations in the femur and acetabulum of subjects with clinically diagnosed cam deformity. The parameterisation was performed for computed tomography scans of 20 patients (10 female and 10 male). Novel quantitative measures of cam deformity were taken and used to assess differences in morphological deformities between males and females.

The parametric surfaces matched the more detailed, segmented hip bone geometry with low fitting error. The quantitative severity measures captured both the size and the position of cams and distinguished between cam and control femurs. The precision of the measures was sufficient to identify differences between subjects that could not be seen with the sole use of 2‐dimensional imaging. In particular, cams were found to be more superiorly located in males than in females.

As well as providing a means to distinguish between subjects more clearly, the new geometric hip parameterisation facilitates the flexible and rapid generation of a range of realistic hip geometries including cams. When combined with material property models, these stratified cam shapes can be used for further assessment of the effect of the geometric variation under impingement conditions.

## 1 INTRODUCTION

Abnormal geometry of the hip joint is associated with femoroacetabular impingement (FAI), which can result in pain and intraarticular damage. Repeated contact between the femoral bone and acetabular rim (typically resulting from flexion and internal rotation) can cause labral pathology and progressive delamination of cartilage.^1^ The damage mechanisms are not fully understood, and it is unclear why some abnormally shaped hips result in pain and damage whilst others do not.^2^

The risk of symptomatic impingement occurring is likely to depend on differences in natural soft tissue shape and quality, the activities performed by individuals, and, as investigated here, the position and shape of bone abnormalities.^2^ Assessment of tissue abnormalities can be challenging, even in the case of radiographs for the analysis of bone, where the projected outline of the structures is relatively clear. Measurements from 2‐dimensional (2D) radiographs are commonly used in the diagnosis of FAI.^3^, ^4^, ^5^ In particular, alpha angles were first described by Nötzli et al^6^ to assess the size of cam deformities (excess bone on the femoral neck), whilst centre edge (CE) and anteversion (AV) angles are used to identify acetabular abnormalities such as pincer impingement (acetabular overcoverage) and dysplasia (undercoverage).^7^ However, such 2D measurements do not capture the full 3‐dimensional (3D) geometry of the hip.^8^ The alpha angle is limited to providing a rough indication of the cam size in a single 2D view^8^, ^9^; there is variation in alpha angle measuring techniques^10^; and high alpha angles have been found in asymptomatic individuals.^11^ It is therefore not a reliable measurement to use to stratify the population by cam type.

There is a recent shift towards conducting clinical research investigating FAI in 3D. Harris et al^12^ generated statistical shape models of hips with and without cams and found noticeable differences between the groups at the anterolateral head‐neck junction. Bouma et al^13^ defined the “omega surface” on a set of example cases to define the region of impingement‐free motion. More recently, Yanke et al^14^ assessed sex differences in cams using computed tomography (CT) scans and found males tended to have cams of larger volume and height than those in female patients. However, the alpha angle remains the most widely used parameter to quantify differences in femoral morphology.

The overall objective of this work was to develop and test a novel method for generating parametric surfaces and 3D severity measures of cam deformity of the hip joint. A successful methodology was considered to be one precise enough to provide some stratification of the population of subjects with cams. The specific aims of this study were as follows:

1. To develop a geometric parameterisation method that generates subject‐specific parametric surfaces to represent morphology of proximal femurs with cams and lunate articular acetabular surfaces, capturing key variations due to cam deformity in 3D.
2. To apply this method across a clinical data set, allowing measurements assessing impingement risk to be obtained, and assess morphological differences between male and female hips diagnosed with cam type impingement.
3. To verify the parameterisation system using shape fitting error assessment between segmented and parametric bone surfaces and comparison of 3D measures with clinical, radiographic measures.
4. To verify the ability of the femoral parameters to capture cam deformities by comparing with non‐cam cases.

## 2 METHODS

Preoperative pelvic CT images (Sensation 16 CT scanner, Siemens, Berlin and Munich, Germany, voxel size: 0.7422 × 0.7422 × 1 mm) of 20 patients (10 males and 10 females) who underwent surgery for cam type impingement were used in this study. Ethical approval (reference MEEC 11‐044) was granted by the faculty research ethics committee at the University of Leeds. Patients included in the study were able to give written, informed consent, were aged 18 years or over (and skeletally mature at the time of scanning) and had a clinical diagnosis of cam FAI with preoperative CT scans readily available. Patients were excluded from the study if they had undergone surgery in the affected hip prior to the CT scan being taken, or in the opinion of the clinical investigator (MB), had an existing condition that would compromise their participation in the study (eg, osteoarthritis).

**Figure 1:**
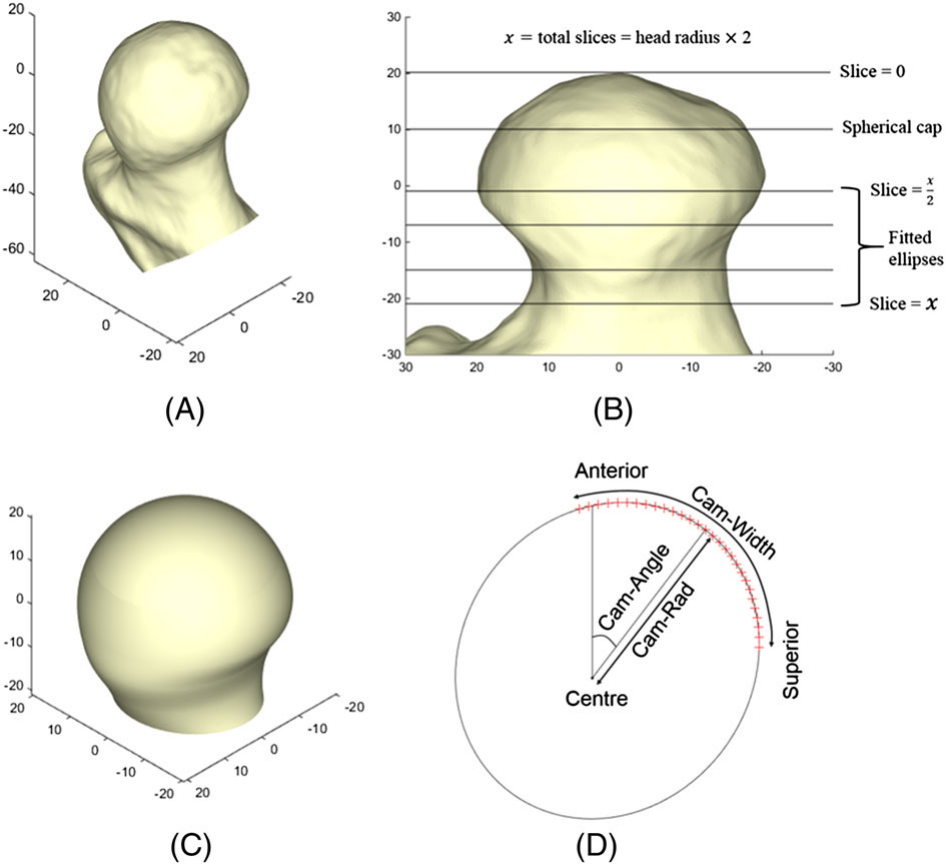
A, Femoral surface segmented from a CT scan (axes in mm). B, Femoral surface aligned to neck axis. Horizontal lines mark the top of the head (where slice counting begins), bottom of the spherical cap region, and the positions of ellipses used for lofting. Ellipses were fitted to vertices on the triangulated surface falling within 2‐mm slices centred on these lines (axes in mm). C, Parametric femoral surface generated by lofting, starting from the bottom of the spherical cap and proceeding through the 4 ellipses (axes in mm). D, Impingement risk measurements shown on a single ellipse; the overall measurements are the mean of each of these measurements across the 2 central ellipses

**Figure 2:**
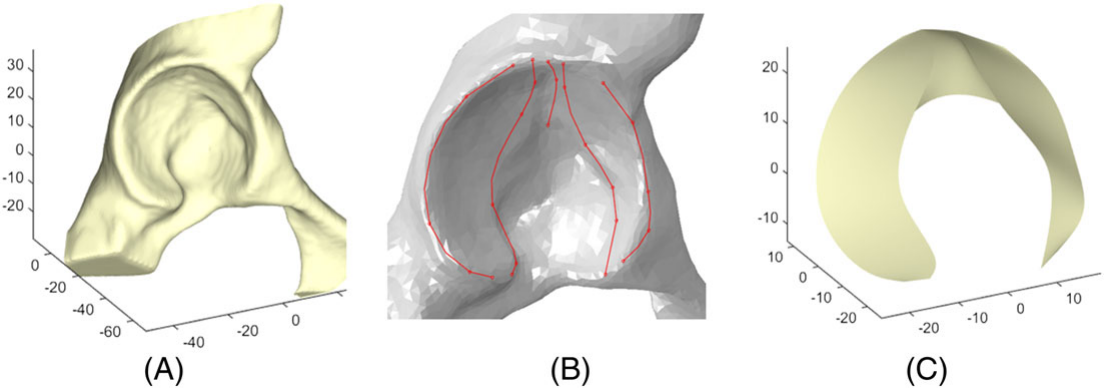
A, Pelvic surface segmented from CT scan (axes in mm). B, Splines fitted to selected nodes on the triangulated surface to capture the lunate surface geometry. C, Parametric acetabular surface generated by lofting through the splines (axes in mm)

Bone surfaces (the proximal femur including the cam deformity and the lunate surface of the acetabulum) were segmented from the CT scans using the image processing software ScanIP (version 7.0, Simpleware Ltd, Exeter, UK) (Figures 1A and 2A). Thresholding was used to isolate the bone, and this mask was then refined with paint tools and smoothing and finally exported as triangulated surface meshes. Parametric surfaces were semiautomatically generated in 2 main steps. The parameters are described in detail in Sections 2.1 and 2.2 for the femoral head and acetabular cavity, respectively. The geometry parameters were systematically extracted from bone surface meshes using a custom‐made code in MATLAB (version R2014b, The MathWorks Inc, Natick, Massachusetts). Parametric surfaces were then automatically generated from these parameters using Python (version 2.7.3) with Abaqus (version 6.14, Dassault Systèmes, Vélizy‐Villacoublay, France). Fitting errors were calculated between segmented and parametric triangulated surfaces at different mesh densities; this is discussed in Sections 2.3 and 4.2.

Measurements were automatically derived from the parameters to indicate the risk of impingement on the basis of 3D hip bone geometry. For comparison, 2D clinical measurements of the same hips were taken from reconstructed radiographs, created by taking averages of CT slices in certain views. Details of this are given in Section 2.4.

To assess the ability of the cam parameters to distinguish between patients diagnosed with cams and those without, we also tested the femoral parameterisation process on an additional 18 control femurs (10 females and 8 males). These patients had undergone CT scans because of hip pain but were not diagnosed with cam impingement. Femoral bones from these patients were segmented and processed with the same methods used for the main patient group.

### 2.1 Femoral parameterisation and severity measurements

First, the head centre was located by fitting a sphere to the nodes on the proximal side of the femoral head. In cases where there was clear external rotation visible in the axial CT view, this was corrected by performing an initial rotation of 20° about the axial axis passing through the centre of the head. This was necessary in 3 of the cam patient femurs and 1 of the control femurs, where the rotations were likely due to the subjects moving from neutral position in the scanner.

All segmented femurs were then rotated to align the neck axis with the vertical image axis (in line with the superior‐inferior axis) using a rotation about the anterior‐posterior axis (Figure 1B). This rotation was based on the assumption that patients' hips were in a neutral position in the CT scans and was chosen to be 40° for all femurs.

In this new orientation, the head centre position and radius were recalculated by again fitting a sphere to the nodes on the proximal head surface. Surface nodes found in 2‐mm thick intervals of the head‐neck region perpendicular to the neck axis were then used to define slices of the proximal femur. The total number of slices, defined from the top of the head to the end of the modelled neck axis, was fixed to be equal to the head radius (rounded to the nearest mm) to ensure that the most distal slice was in a comparable position along the femoral neck for each femur. For example, for a head radius of 25 mm, 25 slices of 2 mm would be used, resulting in a height of 50 mm from the top of the head to the end of the neck. Ellipses were then fitted to the segmented bone surface in a selection of 4 of these slices. The 4 slices were selected automatically by taking a linearly spaced vector with 4 points between $\frac{x}{2}$ and *x*, where *x* was the total number of slices, and then rounding these points to integer values, thus focusing on the femoral neck region.

Parametric surfaces representing the proximal femur (femoral head and neck including cam) were generated using the extracted parameters (Figure 1C). A spherical cap with 25% of the surface area of a whole sphere was generated to represent the proximal head.^15^ Lofting was used to complete the surface, in which a 3D surface was generated by transforming from a starting section shape and orientation to an ending shape and orientation, with intermediate sections defining the shape of the surface as it passes through space. The lofting operation was performed from the circular end of the spherical cap through the 4 ellipses.

Measurements were defined to describe the size and position of the cam to isolate the region with potential to cause impingement. For each slice, the following were defined (Figure 1D):

- Cam‐rad is the greatest planar distance between the head centre and the anterior half of the fitted ellipse, recorded as a percentage of the head radius. This indicates the level of offset between the head and neck in the cam region.
- Cam‐angle describes the position of the point on the ellipse where cam‐rad is defined (ie, where the head‐neck offset is lowest). A zero angle represents an anteriorly centred cam, and greater angles indicate a more superior position.
- Cam‐width is the percentage of the neck circumference whose distance from the head centre is greater than 90% of the distance defined by cam‐rad. Cam‐width therefore indicates the extent to which the circumference of the neck is affected by the cam. The position along the neck where the ellipses were fitted is known from the geometrical parameters.

The average of these measurements from the 2 central loft slices were recorded as the overall Cam‐rad, Cam‐angle, and Cam‐width.

### 2.2 Acetabular parameterisation and severity measurements

Parametric acetabular surfaces were generated by lofting through five 3D spline curves fitted to between 3 and 6 points on the segmented acetabulum (Figure 2A), manually selected to capture the lunate surface (Figure 2B). Each parametric lunate surface (Figure 2C) was generated from a total of 25 nodes selected from the segmented surface, on the outer edges of the acetabulum, the inner edges of the lunate surface, and the superior middle portion of the acetabular cavity.

Two clinically relevant angles, AV and CE angles, were extracted from the 3D spline data representing the acetabula.

**Figure 3:**
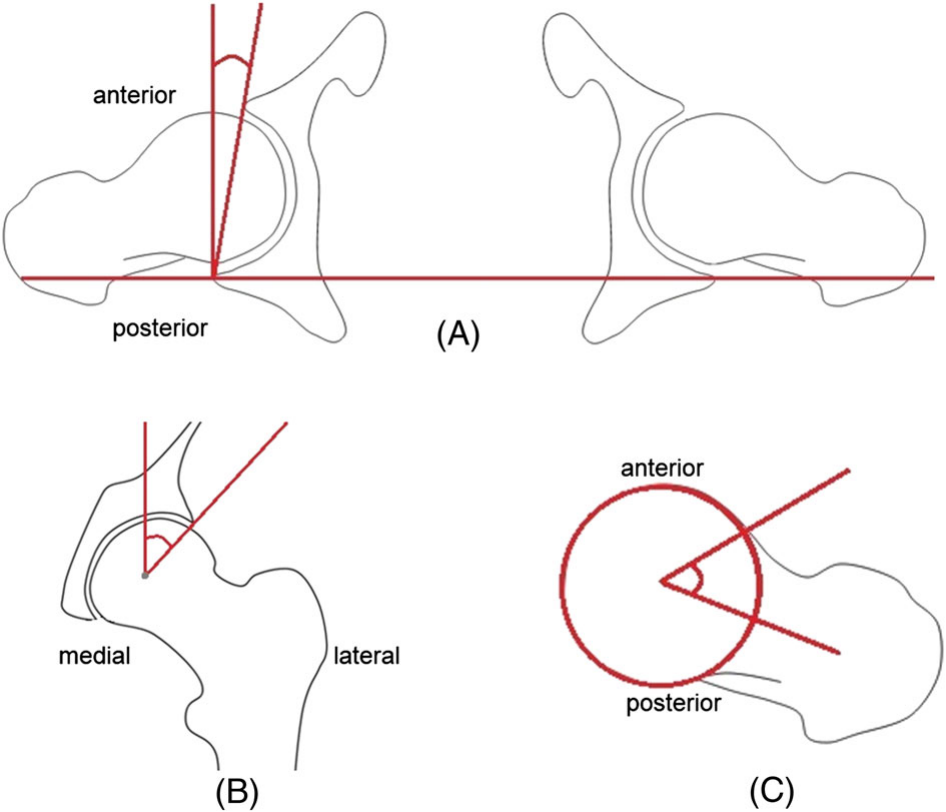
Traditional 2D hip angle measurements; A, anteversion (AV) angle in the transverse plane; B, centre edge (CE) angle in the coronal plane; C, alpha angle can be measured in various 2D views, shown here in the transverse plane

Five AV angles were calculated in the transverse plane as the acute angle between AP‐axis and the line between the most anterior and posterior points on the acetabular rim (Figure 3A). The most anterior and posterior points on the rim were captured by the nodes lying on the 2 outermost splines. The AV angle measurements were taken at different positions along the superior‐inferior axis corresponding to the 5 different nodes defining the outer splines. The mean AV angle from these 5 measurements was taken as the overall 3D measured AV angle, quantifying the amount by which the acetabulum as a whole was anteverted.

Five CE angles were calculated in the coronal plane as the angle between the vertical line passing through the femoral head centre and the line joining the head centre and the edge of the acetabulum (Figure 3B). The most superior node on each spline represented the top edge of the acetabulum. By taking the measurement for each spline, 5 measurements were obtained at different positions along the anterior‐posterior axis. The maximum CE angle from these 5 measurements was taken as the overall 3D measured CE angle, quantifying the most severe overcoverage in the acetabulum.

### 2.3 Fitting error calculations

Fitting errors were calculated in MATLAB from the triangulated surfaces as the root mean squared distance that nodes on the segmented surface in the region of interest had to move to conform to the nearest node on the parametric surface.

A sphere was created in the +CAD module of ScanIP and resampled to the CT scan voxel resolution of 0.7422 × 0.7422 × 1 mm. The femoral parameterisation procedure was applied to this triangulated sphere to obtain the baseline fitting error, ie, the best fitting error that could be achieved using this method at the specified CT resolution.

### 2.4 Two‐dimensional measurements

For each of the 20 patients, 3 reconstructed radiographic views (described in the following paragraphs) were generated by using ImageJ (version 1.49 m, National Institute of Health, Maryland) to rotate the CT slices where necessary and then average the slices of interest to create a simulation of a radiographic view. This process used a fixed protocol and was not subject to interuser variation. OsiriX (lite 32 bit version 6.0.1, Pixmeo, Geneva, Switzerland) was used by a consultant radiologist (PR) to take 2D measurements of alpha, AV, and CE angles from these reconstructed radiographs for each patient. An additional 3 researchers (RJC, ACJ, and MM) repeated the acetabular angle measurements to investigate user variation in reading angles from such radiographs, where outer bone limits can be difficult to assess.

An axial view was created by averaging the transverse CT slices that included the acetabula. The AV angle was measured in this view as the angle between the line perpendicular to the line joining the posterior edges of the acetabula on both sides (ie, the anterior‐posterior axis) and the line joining the anterior and posterior edges of the acetabulum (Figure 3A).

A coronal view was created by rotating transverse slices into the correct orientation and averaging the slices including the relevant femur. Both femurs were included in these images to verify that the vertical axis represented an inferior‐superior axis. The CE angle was measured in this view as the angle between the vertical line through femoral head centre and the line passing through the femoral head centre and the top edge of acetabulum (Figure 3B).

A cross‐table lateral view was created by rotating the femur 15° internally and by a 45° angle from the sagittal plane and averaging CT slices with a view of the medial side of the femur. This view was chosen to measure alpha angles, as it is a standard radiograph and can be readily simulated by rotating CT slices to obtain an image similar in appearance to actual radiographs. Additionally, it has been reported that alpha angles measured in this view correlated well with 3D measured asphericity.^8^ The alpha angle was measured as the angle between the line passing through the femoral neck midpoint and the femoral head centre and the line from the femoral head centre to the anterior point where the femoral head diverges from spherical (Figure 3C). Alpha angles were also measured on the original CT data with oblique axial reconstructions and measurement of the alpha angle at the midpoint image.^6^, ^16^, ^17^

Pearson correlation coefficients (*r*) and linear regressions were calculated between the 3D severity measurements and the 2D measurements, and also between the 2 alpha angle measurements. Independent samples *t* tests were performed to test for differences in the potential impingement severity measurements between males and females (the data were first checked for normality). Independent samples *t* tests were also used to test for differences in the femoral severity parameters between the patient and control femurs (the data were again first checked for normality). All statistics were calculated using the Statistics and Machine Learning Toolbox in MATLAB.

## 3 RESULTS

The data associated with this paper are openly available from the University of Leeds data repository.^18^

Parametric bone surfaces were a good fit to the segmented surfaces, with an average fitting error of 0.57 mm for cam femoral surfaces and 0.85 mm for the acetabular surfaces. The fitting error between the resampled sphere and the parametric sphere was 0.22 mm, representing the best fit that could be obtained with the procedure using the CT scan resolution and converged mesh density.

**Figure 4:**
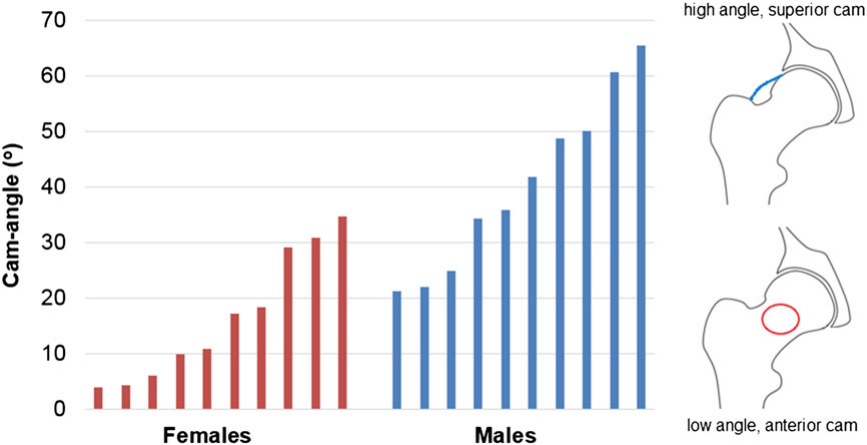
Comparison of cam position in females and males. Cams with a high cam angle are located more superiorly on the neck, shown in the femur diagrams

A wide range of values for the cam severity measurements was found (Table 1). In particular, a statistically significant difference (*P* = 0.0011) was identified showing that the average cam‐angle was higher in male cam patients (mean 40.5°) than female cam patients (mean 16.5°) (Figure 4). The 95% confidence interval for the difference in means between these groups was (11.1°, 36.9°). None of the other cam severity measures and neither of the acetabular angles showed a significant difference between males and females.

**Table 1:** Femoral severity measurements obtained from parameterised surfaces representing the 20 cam and the 18 control femurs

		Cam patient femurs			Control femurs		
		Combined	Females	Males	Combined	Females	Males
Cam‐rad (%)	Range	86.1‐100.4	86.1‐99.6	89.3‐100.4	79.2‐93.9	85.1‐91.1	79.2‐93.9
	Mean ± SD	93.9 ± 4.4	92.7 ± 4.8	95.0 ± 4.0	89.3 ± 3.4	89.1 ± 2.3	89.5 ± 4.6
Cam‐angle (^o^)	Range	4.0‐65.5	4.0‐34.7	21.2‐65.5	2.9‐50.8	2.9‐35.7	5.1‐50.8
	Mean ± SD	28.5 ± 18.2	16.5 ± 11.5	40.5 ± 15.7	19.8 ± 12.5	15.3 ± 10.1	25.3 ± 13.6
Cam‐width (%)	Range	28.0‐67.0	28.0‐67.0	30.5‐60.5	29.5‐100	35.5‐100	29.5‐70.5
	Mean ± SD	47.1 ± 13.4	44.7 ± 14.1	49.5 ± 13.0	56.4 ± 20.9	61.7 ± 24.9	49.7 ± 13.2

**Figure 5:**
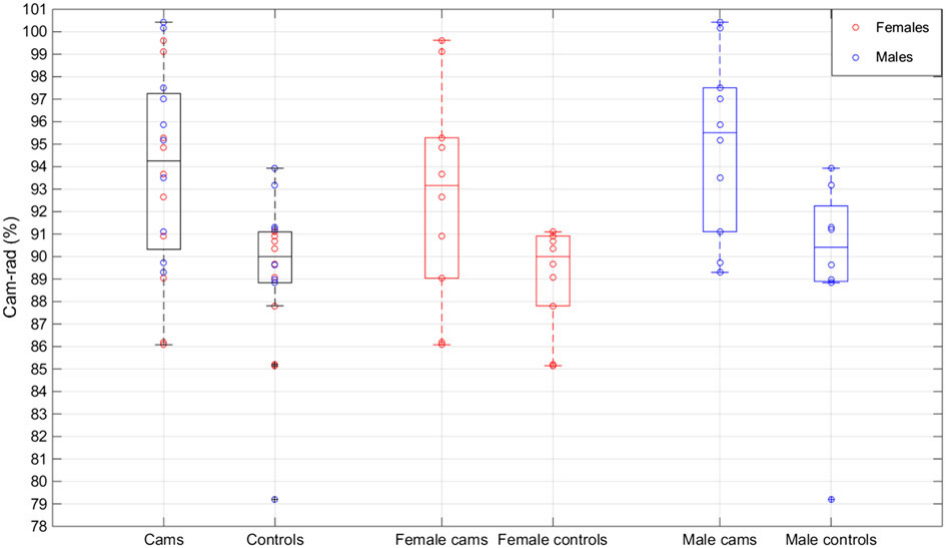
Box and dot plot showing all of the cam‐rad measurements, to aid visualisation of the statistical significance of the differences between the control and cam groups, both overall and in the female and male groups separately

A statistically significant difference (*P* = 0.0014) was identified showing the average cam‐rad was higher in the cam patient group than in the control group, and this was true for both the male and female groups as well as overall (Figure 5). The differences remained significant when outlying cases were removed. No significant difference was identified between the patient and control femurs for the other parameters. The identified difference in cam‐angle between males and females was not present in the control femurs.

**Figure 6:**
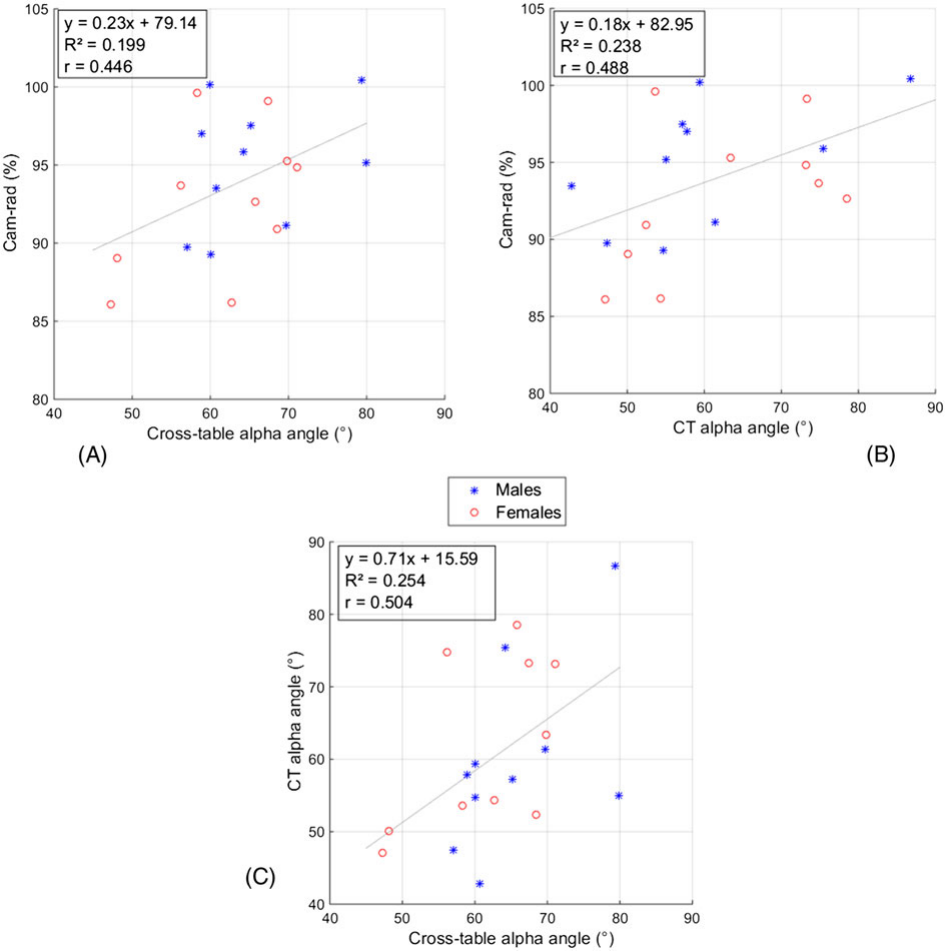
Moderate correlation was found between: A, cam‐rad measurements and the cross‐table measured alpha angles; B, cam‐rad measurements and the CT measured alpha angles; and C, the 2 methods used to measure alpha angles

The 2 alpha angle measurements on the cam patients were only moderately correlated with each other and both were only moderately correlated with the cam‐rad measurements (Figure 6).

**Figure 7:**
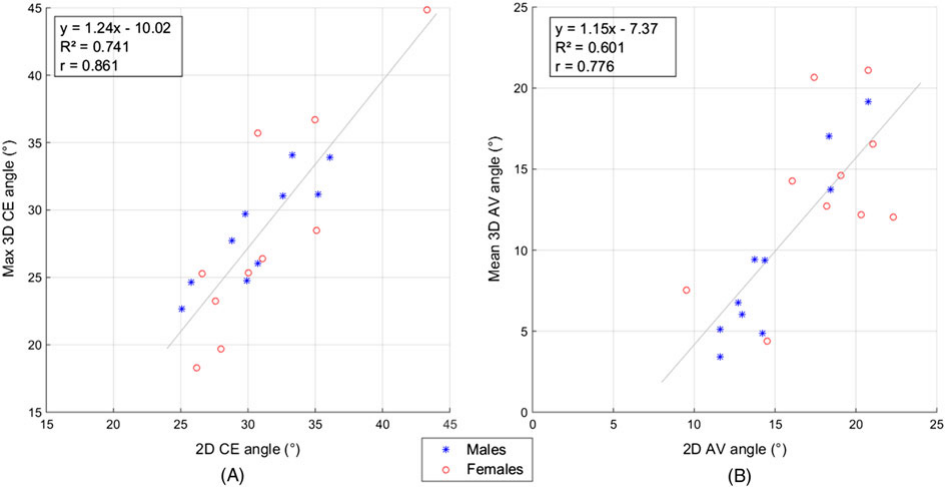
Strong correlation was found between: A, the 2D CE angles and the maximum 3D CE angles; B, the 2D AV angles and the mean 3D AV angles. AV, anteversion; CE, centre edge

The 2D measured CE and AV angles were well correlated with the 3D measured versions (Figure 7). When 3 additional researchers measured the 2D CE and AV angles to investigate interuser variability, the average over all of the femurs of the standard deviation across measurers was 1.97° and 1.90°, respectively.

## 4 DISCUSSION

The aims of this study were to develop a geometric parameterisation tool to capture key hip shape variations in 3D and to use it to assess morphological differences between male and female hips with clinically diagnosed cam type impingement. The novel 3D measurements obtained from the semiautomatic parameterisation system provided additional information on the shape and position of cams not captured by 2D measurements. This allowed differences between the male and female groups to be identified. Male subjects were more likely to have a superiorly located cam (or “pistol grip” deformity), whereas cams in female subjects were more likely to be in an anterior position.

Performing the femoral parameterisation process on a sample of control femurs verified its ability to differentiate between femurs diagnosed with cams and those without. Although control femurs do not have cams, the cam‐angle parameter still detects the region with smallest head‐neck offset. The lack of difference in cam‐angle between males and females in the control group suggests that the more superior location in males of this region may be a specific observation of morphological changes related to cam deformity, rather than being true in general. Since cam‐width is a measure on the basis of the value of cam‐rad, a direct comparison between control and cam groups was less useful. Cam‐width was generally higher in the control group simply because the cam‐rad values were lower.

The average fitting errors between the parametric and segmented geometries were of a similar magnitude to others reported for articular surfaces of the hip approximated by sphere and conchoids^19^ and were smaller than differences between subjects. Good correlation was found between CE and AV angles and the 3D counterparts, providing additional confidence in the 3D representation of acetabular coverage. Comparisons between the 3D cam severity measures and the alpha angle measurements clearly demonstrated the challenges in assessing cam geometry in a 2 dimensional view.^8^, ^9^

### 4.1 Significance

Recently, it has been reported that cams in female patients present in the same location as in males, but the volume and span of the cam was greater in males.^14^ Cam positions have also previously been reported to be anterolateral to anterior in females, compared to lateral to anterior in males.^20^ Cam position was assessed in this study using the greatest radius of the cam (the cam‐angle parameter is defined on the basis of cam‐rad, defined by the region with the lowest head‐neck offset). This allowed the detection of the more generally superior position of the cam in the male patient group and more anterior position in female patient group.

The size of a cam (as measured here by cam‐rad) cannot be easily predicted from a single radiographic alpha angle. It was seen that multiple subjects with very similar alpha angles had quite different 3D severity measures. Alpha angles are dependent upon the view in which they are measured, which is evident here in the differences between the CT and cross‐table alpha angles (Figure 6), and has been reported by others.^8^, ^21^, ^22^ Thus, it is possible for the same alpha angle to be recorded for cams of different sizes and positions. In addition to size of the cam for head‐neck offset, this study also provides a novel method of quantifying the variation in cam position and extent of neck coverage (captured by the cam‐angle and cam‐width measurements). Such information cannot be obtained from an alpha angle measurement. Whilst X‐rays are taken as standard, not all clinics use CT scans, which expose patients to high levels of radiation, or MRI scans, which can be prone to distortion and are less optimal for viewing bone. However, the risk of impingement in different hip morphologies can be more accurately quantified when 3D imaging of the patient is available and this emphasises limitations of relying on radiographs alone. Radiographically, an AP view shows cams in a superior position more clearly, whilst the cross‐table view is more effective for detecting cams in an anterior position. The differences seen between cam positioning in male subjects (more superior) and female subjects (more anterior) suggest that when only 2D imaging is available, the choice of primary radiographic view could be tailored to sex of the patient; cross‐table radiographic views are even more important in females. However, AP view is still essential to identify abnormalities of acetabular morphology such as dysplasia and protrusio acetabuli.

It has been reported that AV angles are usually higher in females whilst CE angles are usually not different between males and females.^7^ This corresponds with the trends found in this study. Further, strong correlation between the 2D measured and maximum 3D measured CE angles (Figure 7A) suggests that the maximum 3D CE angle calculated from the parametric surfaces is a reliable assessment of the overall level of acetabular coverage. Consideration of all 5 CE angles could provide information on the level of acetabular coverage across different positions along the anterior‐posterior axis, with a mean range across all patients for the 5 CE measurements of 15.8°. This information cannot be gleaned when only a radiographically measured CE angle is available. It may be possible to use differences in angles from the splines to indicate regions at higher risk of pincer impingement, although no pincer patients were available for this study. Similarly, strong correlation between the 2D‐measured and average 3D‐measured AV angles (Figure 7B) suggests that the mean 3D AV angles from the parametric surfaces provide a valid indication of the overall level of AV of the hip, and consideration of all 5 AV angles could provide information on the level of anterior acetabular coverage along different axial regions along the superior‐inferior axis. The mean range across all patients for the 5 AV measurements was 7.5°. Again, this variation is information not captured when only a single 2D AV angle is recorded from an axial CT slice.

The low standard deviations between users when additional researchers measured acetabular angles suggest that user variation would have minimal effect on the detected correlations. Interuser variation in alpha angles is likely to be higher, but alpha angles are in any case greatly affected by viewpoint.^8^, ^9^

The automatic method of generating parametric surfaces provides the ability to represent the large variation in hip morphology^23^ across populations. Changes to individual parameters can be used to represent precisely defined, clinically relevant morphological differences. The parametric surfaces could therefore be used in finite element models to assess the effects of morphological changes on contact mechanics, providing potential to further investigate impingement damage mechanisms.

### 4.2 Limitations and challenges

The parameterisation system depends on segmentation of bone from 3D images and on the assumption that hips are orientated neutrally in anatomical planes. In 3 cam femurs and 1 control femur, this was seen in the axial view to be untrue, and an additional rotation was used to align them approximately into a neutral rotation. Without this correction, the fitting errors for these cases were noticeably larger (>1 mm). Further, the neck axis was assumed to be at 40° to the superior‐inferior axis for all femurs since it was not possible to measure the femoral neck shaft angle given the field of view in the scans, and defining the neck axis for each femur presented a significant challenge because of the abnormal morphology resulting from the cam deformities. The value of 40° was therefore chosen to automate the process as it appeared in all cases to orientate the neck axis approximately vertically, as seen in Figure 1B. Sensitivity tests revealed that varying this angle affected cam parameters because the ellipse was captured on a different plane through the neck. However, even when varying the angle by 25%, these differences were generally less than the differences observed between individuals, so the cam‐rad measure was still capable of detecting differences between cams of different severities. The identified difference between cam position in males and females was also still apparent.

The segmentation of bone surfaces from CT scans followed a set protocol and user variability would be unlikely to cause more than minor differences in final parametric models. Once bone surfaces have been segmented from CT scans, the method is mostly automatic. The femoral parameterisation and severity measurements assessing the cam are obtained fully automatically through scripts. The acetabular parameterisation requires manual intervention (including some expert adjustments) to select nodes on the acetabulum to obtain an optimum fit, necessary because subject‐specific irregularities in acetabular rim shape are not well captured using standard shapes. The measured angles were calculated from nodes around the rim, which were the most straightforward to place, limiting the effect of this variation on acetabular severity measurements. Whilst the process would need further refinement and optimisation to be used as a clinical tool, particularly on the acetabular side, which has not been tested on pincer patients, it is presently capable of capturing complex hip geometry in a finite number of simple geometric parameters. Variations of both surfaces with alternative parameters can be generated automatically, and it is possible to vary each parameter separately.

Fitting errors between triangulated surfaces are higher when coarse surface meshes are used because the distance from each node on a segmented surface to the nearest node on the equivalent parametric surface may be higher than the true distance between the surfaces at that point. Therefore, the mesh densities of both the parametric and segmented surfaces were iteratively increased until the fitting errors converged so that the reported fitting errors (and extracted parameters) were limited by the resolution of the original CT scans rather than the mesh density. This occurred when mesh densities were such that nodes on each surface were spaced at distances of around 0.25 to 0.3 mm.

Preliminary tests found that using 4 ellipses was sufficient to capture the geometry with a similar fitting error to that found when all slices below the middle slice were included. Using more slices resulted in an uneven surface because of the reduced distance between ellipses lofted through, whilst using less than 4 ellipses leads to considerably poorer fits between the parametric and segmented surfaces. Because there is some noise (as the best possible fitting error was seen to be 0.22 mm), the measures describing cam severity are relative rather than absolute. The highest value of cam‐rad, 100.4%, should be interpreted as indicating a particularly severe cam where there is very little head‐neck offset, rather than suggesting the cam radius is literally greater than that of the head. Cam‐rad is sensitive to the position of slices used for lofting, which is why the method for choosing number of slices was standardised as equal to the head radius and linearly spaced slices were chosen automatically. Whilst a more accurate value for cam‐rad could be obtained from the full segmented surface, the described method allows all the severity measurements to be derived automatically using only the geometrical parameters describing the bone shape, and the precision was sufficient to demonstrate differences between 2 population groups.

### 4.3 Conclusions

This study has demonstrated that it is possible to represent segmented geometry of the acetabular cavity and proximal femur bone with a cam deformity, using a small number of parameterised curves and achieve a low overall fitting error. By adjusting the parameters, variations of the parametric surfaces can be automatically generated. The novel 3D cam severity measurements obtained from the parameterised geometry provide a systematic method of assessing impingement risk and impart more information on the shape and position of cams when compared with using only radiographic measurements, which may not give a good indication of the full extent and exact location of bony abnormalities. The potential severity, ie, risk of impingement, on the femoral side can be assessed by the cam size resulting from head‐neck offset (captured by cam‐rad) in a manner that is not dependent on a 2D view. Combining this with the position and extent of the cam (captured by cam‐angle and cam‐width), and with the acetabular severity measurements, could allow impingement to be predicted on the basis of bone shape in different scenarios, although there are other factors that play a role in its severity, such as patient activity level and vulnerability of the labrum and articular cartilage to injury. The measures developed here also allowed investigation of the differences in cams between males and females, which showed that cams in males are more likely to be superiorly located.
